# Investigating the Potential of Propranolol as an Anti-Tumor Agent in Colorectal Cancer Cell Lines

**DOI:** 10.3390/ijms26157513

**Published:** 2025-08-04

**Authors:** Shiekhah Mohammad Alzahrani, Huda Abdulaziz Al Doghaither, Hind Ali Alkhatabi, Mohammad Abdullah Basabrain, Peter Natesan Pushparaj

**Affiliations:** 1 Biochemistry Department, Faculty of Science, King Abdulaziz University, Jeddah 21589, Saudi Arabia; salzahrani1329@stu.kau.edu.sa; 2 Institute of Genomic Medicine Sciences, King Abdulaziz University, Jeddah 21589, Saudi Arabiapnatesan@kau.edu.sa (P.N.P.); 3 Department of Biological Science, College of Science, University of Jeddah, Jeddah 21959, Saudi Arabia; 4 Department of Medical Laboratory Technology, Faculty of Applied Medical Sciences, King Abdulaziz University, Jeddah 21589, Saudi Arabia

**Keywords:** repurposing drug, propranolol, colorectal cancer, migration, reactive oxygen species, apoptosis, cell cycle, capecitabine, combination index

## Abstract

The incidence and mortality of colorectal cancer (CRC) have increased globally. Several therapeutic approaches have been suggested to address this health issue, in addition to classical methods. Propranolol (PRO) is a beta-blocker that was repurposed to treat infantile hemangiomas, and its anti-tumor activity has been reported. This study aimed to investigate the effects of PRO in a panel of CRC cell lines and its potential impact when combined with chemotherapy. The effects of PRO on cell cytotoxicity, cell morphology, colony formation, cell death induction, cell cycle, mitochondrial and intracellular reactive oxygen species (ROS), and migration were measured in all cells. CompuSyn software was utilized to assess the possible synergistic or additive interaction in the combined treatment. The results showed that PRO suppressed cell proliferation, altered cell morphology, inhibited colony formation, induced apoptosis, altered cell cycle and ROS generation, and inhibited the migration of treated cells in a cell-type-specific, time-dependent, and dose-dependent manner compared with the control. HT-29 was the most sensitive cell line to PRO in terms of cytotoxicity, apoptosis, cell cycle arrest, and ROS generation, while SW-480 was the most sensitive in terms of migration inhibition. Moreover, the PRO and capecitabine combination exhibited a synergistic effect and induced mitochondrial apoptosis in metastatic CRC cells. The data suggest that PRO could be a promising adjuvant therapy for primary and advanced CRC. This study identified variations between CRC cell lines in response to PRO, which may be related to their genetic and epigenetic differences. In addition, the findings highlight the potential of combination strategies to improve therapeutic outcomes in metastatic CRC.

## 1. Introduction

PRO is a non-selective beta-blocker (β-B) that is clinically prescribed to treat cardiovascular diseases [1,2]. However, it has attracted considerable attention for its potential as an anti-tumor therapy since it has been approved by the Food and Drug Administration (FDA) as a first-line treatment for infantile hemangioma [1,3]. This treatment strategy is known as a repurposing drug, a therapeutic approach utilized in clinical settings that entails the use of current FDA-approved medications for purposes different from their primary indication because of their proven safety and low cost [4,5]. Hence, PRO has been investigated in preclinical and clinical studies as a repurposed drug for cancer treatment, including CRC [6,7,8,9,10,11]. Accumulating evidence has demonstrated that PRO exerts anti-tumor effects in oncology by modulating the adrenoreceptor signaling pathway [12]; suppressing tumor proliferation; and modulating apoptosis [10,13,14], angiogenesis [15,16], immune response [11], and the tumor microenvironment (TME) [17]. Moreover, PRO improves the efficacy of classical chemotherapeutics, immunotherapy, and anti-diabetic agents [1,17,18,19].

In this study, we focused on CRC, owing to its aggressive features [20], which are accompanied by the accumulation of various genetic and epigenetic alterations during tumor development [21] that improve drug resistance [22]. Few studies have reported the anti-tumor effects of PRO therapy in preclinical and clinical settings with respect to CRC, including the above-mentioned reports. For instance, a novel study reported that PRO induced ferroptosis as a monotherapy and displayed a synergistic effect when conjugated to capecitabine in HT-29 cells [19]. A randomized controlled trial reported that PRO activated intratumoral CD8+ T cells and decreased the expression of phosphorylated ERK in CRC patients [11]. Moreover, a pilot clinical trial found that the combination of PRO and etodolac treatment significantly decreased the risk of CRC recurrence at five years [23]. In addition, a review article suggested that PRO could also be implemented as a cardioprotective agent against the potential cardiac complications of chemotherapy and radiotherapy, especially in elderly patients with cancer [24], and may be used as a promising novel adjuvant/co-adjuvant therapy for CRC [2].

There is little preclinical evidence of PRO’s potential therapeutic impact on CRC, particularly in terms of investigating the variations in responses between CRC cell lines in cell proliferation, cell survival, apoptosis, cell cycle, migration, and oxidative stress after treatment. Therefore, this in vitro study investigates the effectiveness of PRO in primary and advanced CRC cell lines. Using a panel of human CRC cell lines, we assessed cell cytotoxicity, cell survival, the alteration of several biochemical parameters, and the anti-migration role of PRO.

In a previous novel study, we evaluated the anti-cancer activity of combing PRO with capecitabine (CAP), a chemotherapeutic agent, in the primary CRC cell lines (HCT-116 and HT-29), which revealed a promising finding [19]. Therefore, the current research also aimed to study the effect of this combination on a preclinical model of the advanced stage of CRC using the SW-620 cell line. Hence, this work will outline the possibility of the efficacy of this new dual therapy on a metastatic CRC model that was not determined previously.

## 2. Results

### 2.1. PRO Induced Cytotoxicity in a Cell-Type-Specific and Dose-Dependent Manner Using Trypan Blue Exclusion Assay

**Figure 1:**
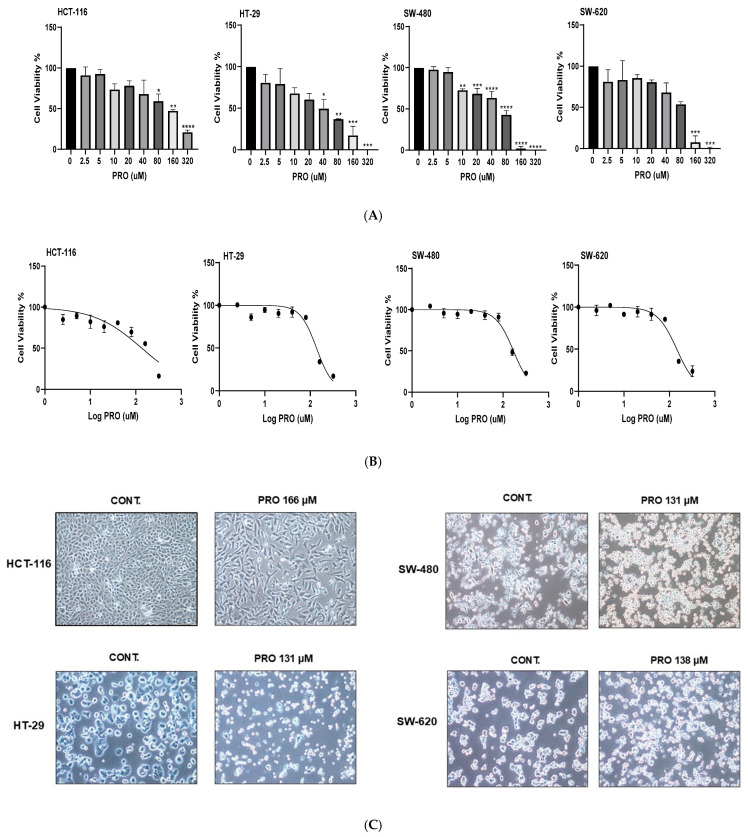
Antiproliferative effects of PRO on CRC cell lines using Trypan blue exclusion and MTT assays. (**A**) Cell viability percentages of PRO-treated cells after 24 h using the Trypan blue exclusion assay. (**B**) Representative dose–response curves for PRO in CRC cells at a density of 10 × 10^3^ cells/well for 24 h using the MTT assay. (**C**) Morphological appearance of CRC cells after PRO treatment. Images were captured using a light inverted Nikon microscope at 10× magnification power. * *p* < 0.05, ** *p* < 0.01, *** *p* < 0.001, and **** *p* < 0.0001.

As shown in Figure 1A, PRO exerted concentration-dependent antiproliferative effects on all tested CRC cells at 24 h. The viability of the treated cells decreased with increasing PRO concentrations. However, significant variability in PRO sensitivity was observed across the cell lines. According to the Trypan blue test, the most sensitive cell line to PRO was SW-480, with a significant reduction in cell viability starting at a concentration of 10 µM. HT-29 cells showed moderate sensitivity to PRO, with a significant value starting at a concentration of 40 µM. Conversely, HCT-116 and SW-620 cells showed the least sensitivity, with significant values starting at concentrations of 80 and 160 µM, respectively. However, the effect of PRO was limited (not significant) when tested at 2.5, 5, 10, 20, and 40 µM on HCT-116 cells and at 2.5, 5, 10, and 20 µM PRO on HT-29 cells. The cell viability of the SW-480 cell line was not significantly changed when exposed to PRO at 2.5 and 5 µM. In addition, the SW-620 cell line also displayed non-significant responses to PRO at 2.5, 5, 10, 20, 40, and 80 µM. These results show that PRO induced cytotoxicity in all human CRC cell lines in a cell-type-specific and dose-dependent manner.

### 2.2. PRO Induced Cytotoxicity in a Cell-Type-Specific, Dose-Dependent, and Time-Dependent Manner Using the MTT Assay

In the first experiment using different cell densities, the results showed increased PRO IC_50_ values in each cell line with increasing cell densities within 24 h, as shown in Table 1. HT-29 was the most sensitive to PRO, with lower IC_50_ values, whereas HCT-116 was the least sensitive, with higher IC_50_ values across cell densities. SW-480 had a moderate sensitivity to PRO. SW-620 displayed a wobbling response to PRO at cell densities of 10, 15, and 20 × 10^3^ cells but showed steady responses at 2 and 5 × 10^3^ cells.

In the second experiment (over various incubation times), the results showed a remarkable reduction in the IC_50_ values of PRO with increasing exposure time at a density of 10 × 10^3^ for each cell line (Table 2). An exposure time of 6 h achieved the highest IC_50_, whereas 72 h achieved the lowest value. The results also indicated time-dependent antiproliferative effects (6, 24, 48, and 72 h). The dose–response curves for PRO in CRC cells at a density of 10 × 10^3^ cells/well after 24 h of treatment are shown in Figure 1B.

### 2.3. PRO Modified Cell Morphology

As shown in Figure 1C, the cell morphology of the PRO-treated cells under the microscope clearly demonstrated modifications when compared with untreated cells, such as changes in the cell membranes, reduced cell confluency, and the presence of apoptotic bodies.

### 2.4. PRO Inhibited Cell Colony Formation

**Figure 2:**
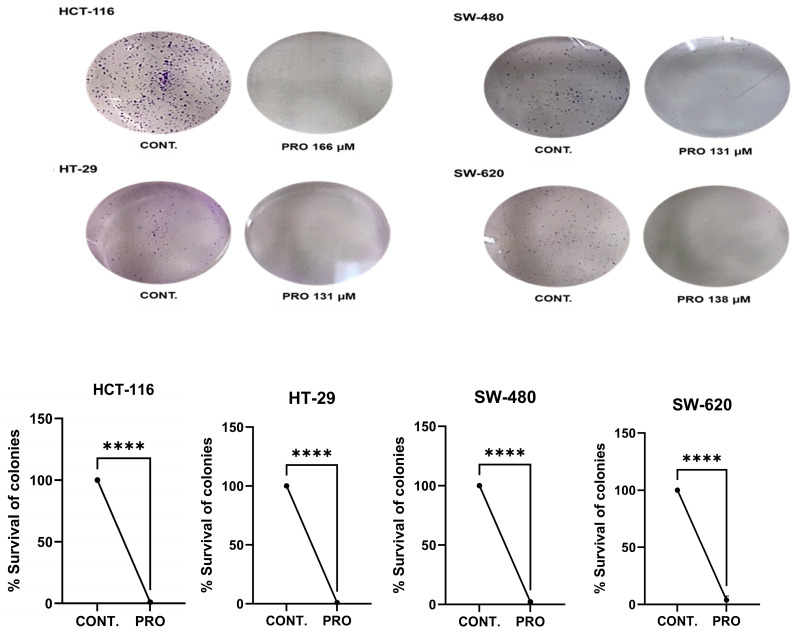
PRO inhibits colony formation in CRC cell lines. The top panel shows representative images of the colony formation assay for CRC cells. The bottom shows the % of colonies in the PRO group versus the control. Comparisons of means were made using unpaired Student’s *t*-tests in GraphPad Prism 9.5.1. **** *p* ≤ 0.0001.

Within the tested IC_50_ concentrations, PRO caused a massive decline in the ability of CRC cells to return to their proliferation capacity compared with the control after replacing the PRO treatment with drug-free media (Figure 2). The PRO groups formed fewer and smaller colonies (**** *p* ≤ 0.0001 vs. control).

### 2.5. PRO Induced Apoptosis in CRC Cell Lines

**Figure 3:**
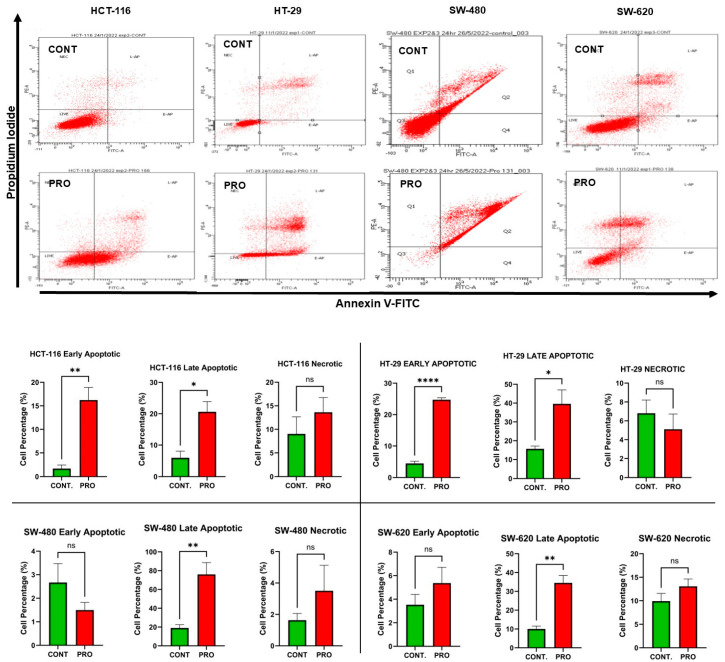
Detection of apoptosis in CRC cell lines after PRO treatment using Annexin V-FITC/PI assay and flow cytometry. The top panel shows representative images of the flow cytometric analysis. The four quadrants of the flow cytometry images indicate the following: LIVE, healthy cells; E-AP, early apoptotic cells; L-AP, late apoptotic cells; NEC, necrotic cells. The bottom side presents the percentages of the early apoptotic, late apoptotic, and necrotic stages of the control and PRO groups in the CRC cell lines. Differences were tested using unpaired Student’s *t*-tests in GraphPad Prism 9.5.1. ns = non-significant value (*p* > 0.05), * *p* ≤ 0.05, ** *p* ≤ 0.01, and **** *p* ≤ 0.0001.

The analysis showed differences in the induction of apoptosis between the CRC cell lines after PRO therapy. PRO significantly increased the percentage of cells at the early apoptotic stage in the HCT-116 cell line (mut *PIK3CA* and mut *K-RAS*), with values of 1.70% and 16.22% for the control and PRO (** *p* ≤ 0.01), respectively. In addition, PRO induced significant apoptosis in the late stage of these cells (* *p* ≤ 0.05) with a percentage of 20.61% versus the control group (5.93%), and no necrotic cell death was detected (Figure 3). By contrast, the data demonstrated that PRO induced significant apoptosis at the early apoptotic stage in HT-29 cells (mut *PIK3CA*, mut *TP53,* and mut *B-RAF*), with values of 4.50% and 24.73% for the control and PRO (**** *p* ≤ 0.0001), respectively. The percentage of cells in the late apoptotic stage significantly increased with PRO (39.60% vs. control 15.66%, * *p* ≤ 0.05), and no necrosis was induced (Figure 3).

In the SW-480 cell line (mut *TP53* and mut *KRAS*), there was no significant change at the early apoptotic stage, whereas late apoptosis significantly increased in the PRO group (76.06% versus control 19.03%, ** *p* ≤ 0.01), and no necrosis was detected. For the SW-620 cell line (mutated *TP53* and mutated *KRAS*), only late apoptosis was induced after PRO therapy (34.53% versus control 10.03%, ** *p* ≤ 0.01) (Figure 3).

### 2.6. Effect of PRO on the Distribution of Cell Cycle Phases

**Figure 4:**
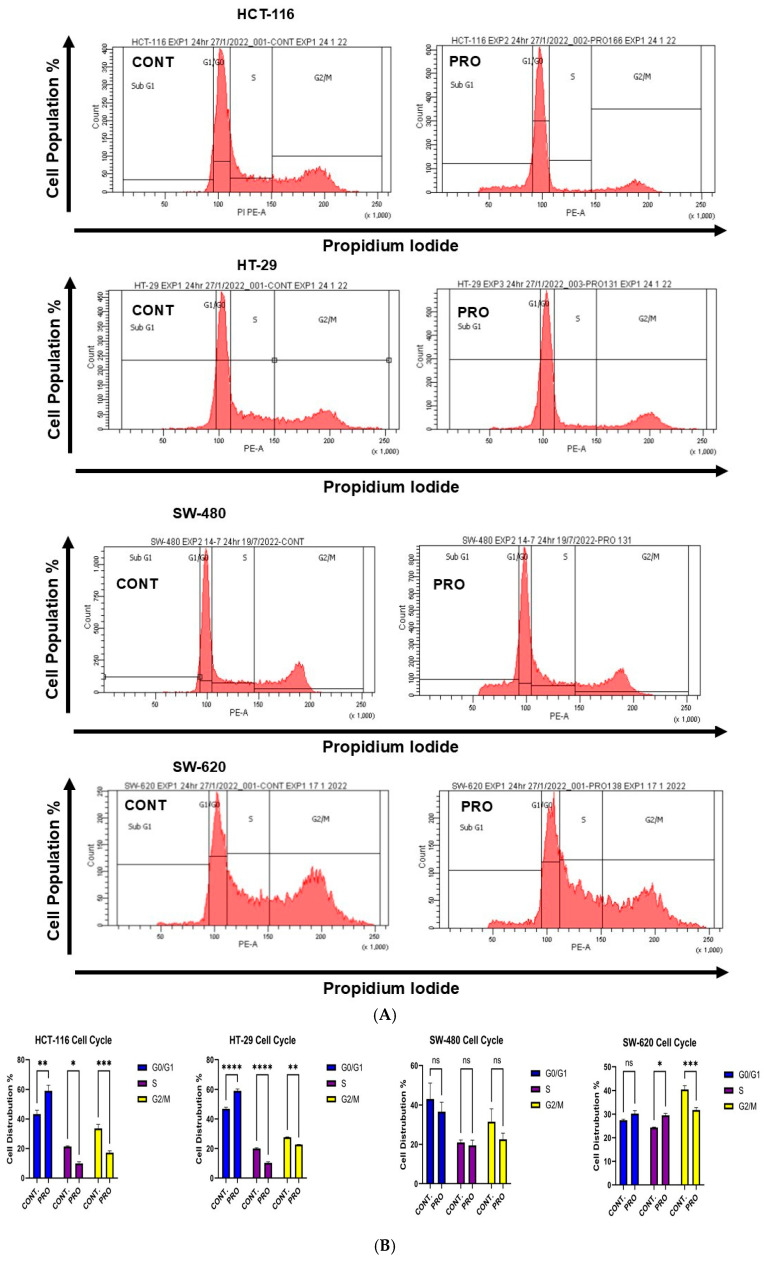

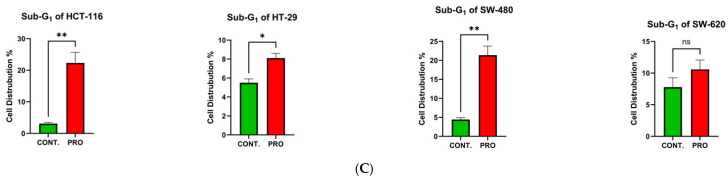
Analysis of the cell cycle in CRC cell lines after treatment with PRO using PI dye and flow cytometry. (**A**) Representative images of flow cytometric analysis of the cell cycle phase distribution in CRC cells. The cell cycle phases in the images are as follows: Sub-G1 phase = cells undergoing apoptosis; G1/G0 phase = growth 1 phase; S phase = DNA synthesis phase; and G2/M phase = growth 2 /mitotic phase. (**B**) Percentages of cells in the G1/G0, S, and G2/M phases of the PRO group versus the control. Comparisons of means were made using a two-way analysis of variance (ANOVA) test in GraphPad Prism 9.5.1. (**C**) Percentage of cells in the sub-G1 phase of the PRO group versus the control. Differences were tested using unpaired Student’s *t*-tests in GraphPad Prism 9.5.1. ns = non-significant value (*p* > 0.05), * *p* ≤ 0.05, ** *p* ≤ 0.01, *** *p* ≤ 0.001, and **** *p* ≤ 0.0001.

In HCT-116 cells, PRO caused arrest at the G0/G1 phase (** *p* ≤ 0.01) by increasing the cell population to 59.06% compared with the control (43.32%). Moreover, the cell proportions decreased at both the S and G2/M phases, with percentages of 9.90% and 17.13%, respectively. By contrast, PRO affected the cell cycle of HT-29 cells by arresting the cells at the G0/G1 phase (**** *p* ≤ 0.0001) and increasing the cell population to 59.00% compared with the control (46.90%). The cell population decreased in the S and G2/M phases compared with the control (Figure 4B). As illustrated in Figure 4B, applying PRO therapy on SW-480 did not significantly alter cell cycle phases. For SW-620 cells, PRO stimulated arrest at the S phase by accumulating cells to 29.51% versus the control value (24.33%) and reduced cells at the G2/M phase (*** *p* ≤ 0.001 versus control) (Figure 4B). In addition, the cell cycle analysis in the sub-GI phase showed that applying PRO to HCT-116, HT-29, and SW-480 cells increased the cell percentage compared with the control (Figure 4C).

### 2.7. Effect of PRO on the Generation of Intracellular ROS

**Figure 5:**
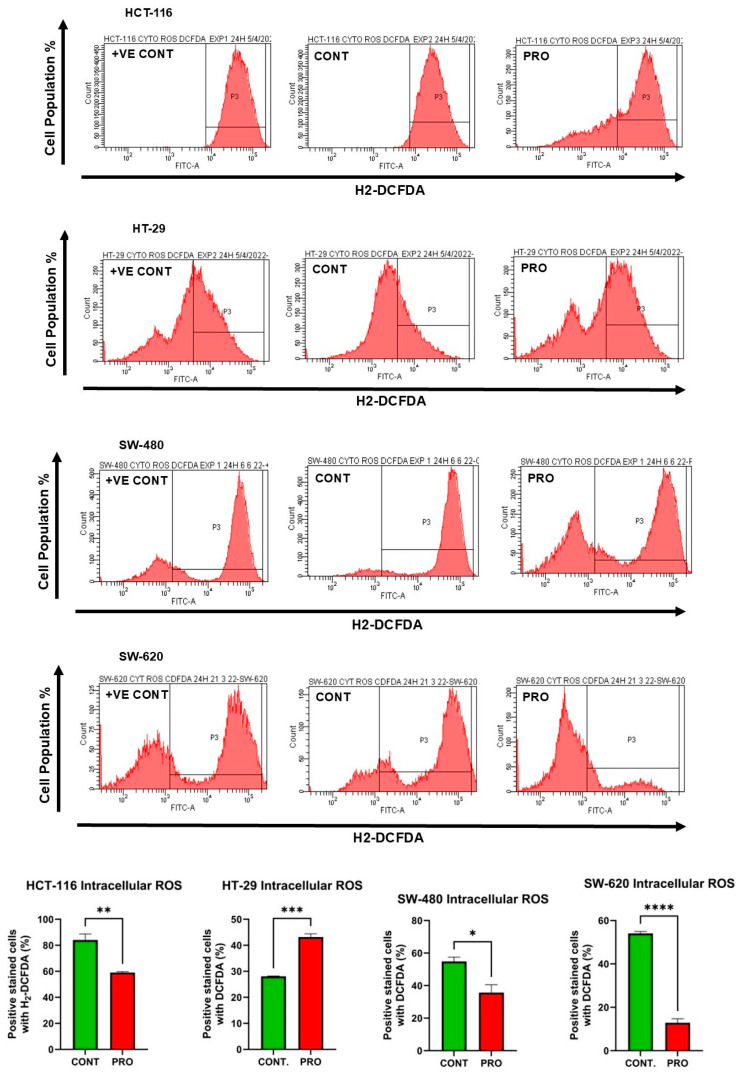

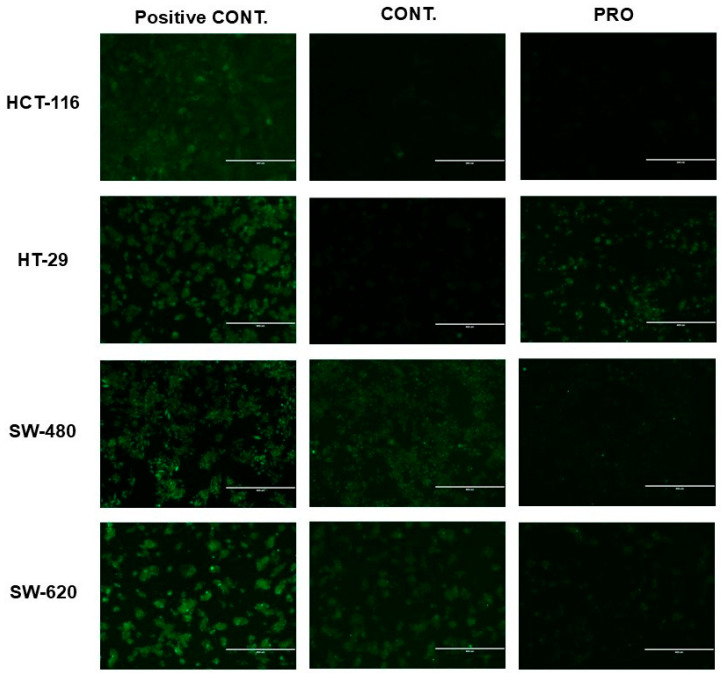
Measurement of intracellular ROS levels in CRC cell lines after treatment with PRO using H2DCFDA dye and flow cytometry. The above side depicts images of the flow cytometric analysis. The middle presents the percentage of CRC cells stained with H2DCFDA dye in the control and PRO groups. Differences were tested using unpaired Student’s *t*-tests in GraphPad Prism 9.5.1. The bottom side shows fluorescent images of intracellular ROS in CRC cells after treatment with PRO, using H2DCFDA dye and an EVOS FL fluorescence microscope. The images of HCT-116 cells were captured at a magnification power of 20×, while those of the other cells were taken at 10× power. ns = non-significant value (*p* > 0.05), * *p* ≤ 0.05, ** *p* ≤ 0.01, *** *p* ≤ 0.001, and **** *p* ≤ 0.0001. +VE CONT, positive control group treated with 10× capecitabine (Sigma-Aldrich, St. Louis, MO, USA).

In HCT-116 cells, the level of intracellular ROS was reduced in the PRO group to 59.06% compared with the control (84.15%; ** *p* ≤ 0.01). For HT-29, flow cytometry analysis showed an increase in intracellular ROS levels to 43.16% for PRO versus untreated cells (28.05%, *** *p* ≤ 0.001) (Figure 5). In addition, the analysis demonstrated a significant reduction in intracellular ROS levels in SW-480 and SW-620 cells after PRO treatment. The values of intracellular ROS were 35.70% versus the control (54.86%) and 12.86% versus the control (54.06%) for SW-480 and SW-620 cells, respectively (Figure 5). Moreover, the intensity of green fluorescence is proportional to the amount of intracellular ROS present in the sample. In Figure 5, the intracellular ROS fluorescent images shows that only HT-29 cell line observed a higher green fluorescence in PRO-treated group versus the control. This observation reflects PRO’s impact in inducing greater cellular oxidative stress and apoptosis through the oxidation of H2DCFDA dye into DCF via an excessive level of various intracellular ROS, resulting in brighter green images in PRO group compared to control. By contrast, the PRO-treated groups in the HCT-116, SW-480, and SW-620 cell lines did not generate high green fluorescence compared to controls, indicating that intracellular ROS did not implicate in the oxidative stress induced by PRO. Additionally, the examination of fluorescent images revealed similarity with the flow cytometric data.

### 2.8. Effect of PRO on the Generation of Mito ROS

**Figure 6:**
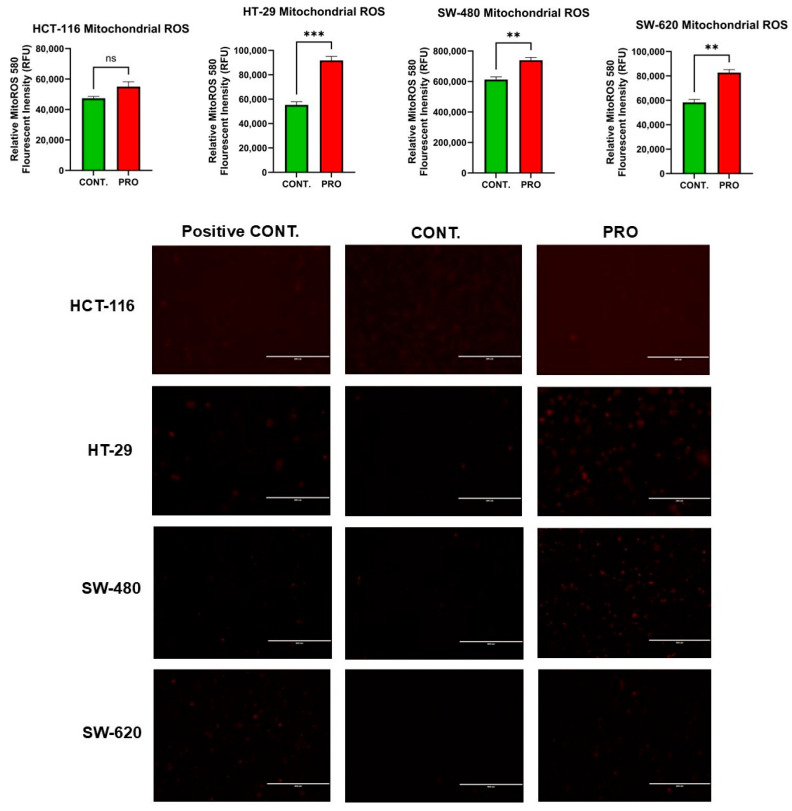
Measurement of Mito ROS generation in CRC cell lines after treatment with PRO using MitoROS 580 dye and a fluorometric technique. The above side is the relative fluorescent intensity (RFU) of the CRC cells for the PRO group versus the control. Differences were tested using an unpaired Student’s *t*-test in GraphPad Prism 9.5.1. The lower panel shows fluorescent images of Mito ROS in CRC cells after treatment with PRO using MitoROS 580 dye and a fluorescent microscope. Images of HCT-116 and HT-29 cells were captured at a 20× magnification, while those of SW-480 and SW-620 were taken at 10× power using an EVOS FL fluorescent microscope. ns = non-significant value (*p* > 0.05), ** *p* ≤ 0.01, and *** *p* ≤ 0.001. CONT, untreated group; +VE CONT, positive control group treated with 10× PRO.

The results for HCT-116 cells indicated that PRO therapy did not affect the formation of Mito ROS, as shown in Figure 6. However, PRO therapy notably elevated Mito ROS formation in HT-29 cells (*** *p* ≤ 0.001). By contrast, a remarkable increase in Mito ROS generation fold change was observed after PRO treatment in SW-480 cells compared with the control. Likewise, using PRO treatment of SW-620 cells had a similar effect on Mito ROS generation as it did on SW-480 cells (** *p* ≤ 0.01). Moreover, the intensity of red fluorescence is proportional to the amount of mitochondrial superoxide present in the sample. In Figure 6, the Mito ROS fluorescent images showed that the HT-29, SW-480, and SW-620 cell lines observed high red fluorescence in PRO-treated groups versus controls with different ranges. This observation reflects PRO’s impact in inducing mitochondrial oxidative stress and apoptosis through the oxidation of MitoROS 580 dye in the mitochondria selectively via an excessive level of mitochondrial ROS (superoxide), resulting in brighter red images in PRO groups compared to controls, while HCT-116 did not generate a significant level of mitochondrial superoxide versus the control. Additionally, the examination of Mito ROS fluorescent images revealed similarity with the fluorometric data.

### 2.9. PRO Inhibited Cell Migration in Cell-Type-Specific and Time-Dependent Manner

**Figure 7:**
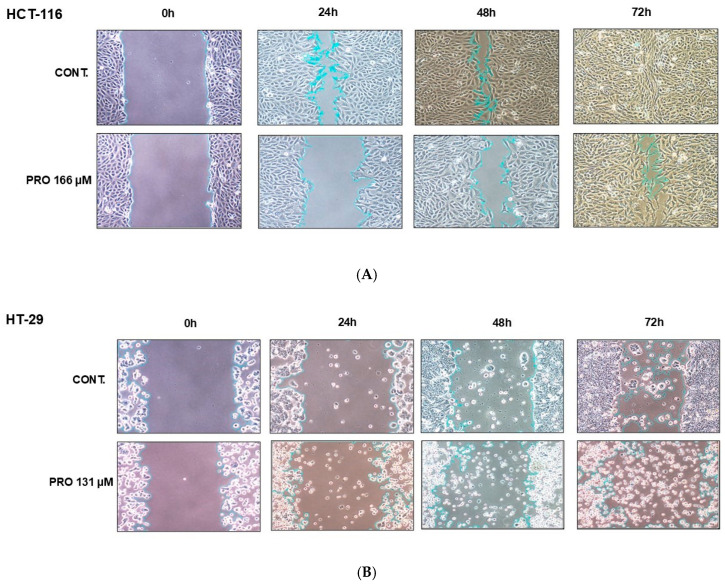

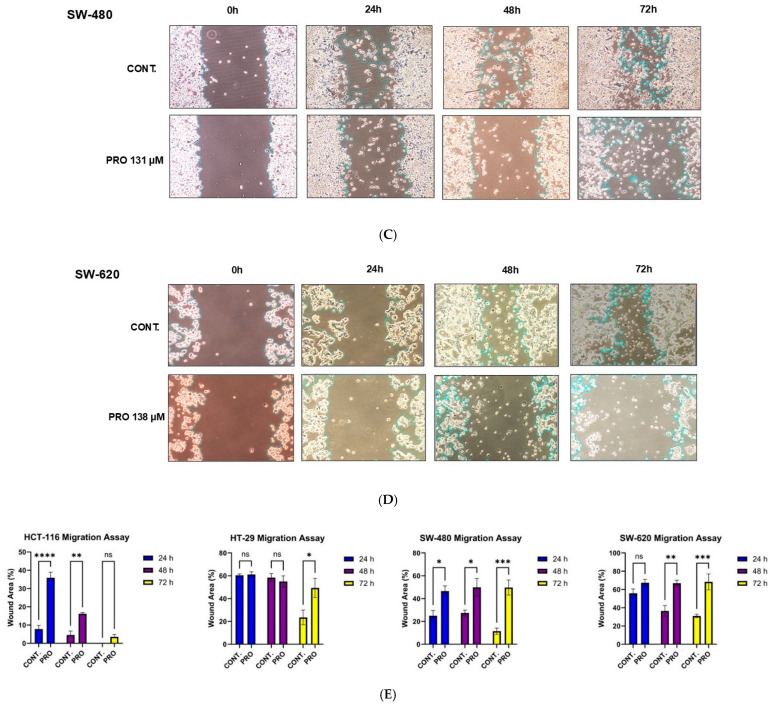
Wound healing assay for CRC cell lines after PRO treatment. (**A**–**D**) Representative images of the migration of CRC cells at 0, 24, 48, and 72 h. Images were taken using an inverted microscope at 10× magnification. The gap area was drawn and then calculated using Fiji (ImageJ version 2.9.0) for each time endpoint. (**E**) Differences in the gap area % of the PRO group versus control among the time intervals (0, 24, 48, and 72 h) are expressed as the mean of three independent experiments ± standard error of the mean (n = 3 ± SEM). Comparisons of means were made using a two-way analysis of variance (ANOVA) test in GraphPad Prism 9.5.1. ns = non-significant value (*p* > 0.05), * *p* ≤ 0.05, ** *p* ≤ 0.01, *** *p* ≤ 0.001, and **** *p* ≤ 0.0001.

Exposure to PRO significantly reduced the migration of HCT-116 cells at 24 and 48 h; however, no significant change was observed at 72 h compared with control cells. For HT-29, PRO significantly altered cell migration capacity at 72 h. The migration ability of SW-480 cells was significantly inhibited by PRO treatment at all time intervals, whereas in SW-620 cells, the inhibition of migration potency was significant at 48 h and 72 h (Figure 7A–E). HT-29, SW-480, and SW-620 cells tended to die and float in the PRO groups at 48 and 72 h, as shown in Figure 7B–D. Analysis of migration demonstrated that PRO reduced the migration of CRC cell lines in a cell-type-specific and time-dependent manner.

### 2.10. Propranolol Synergizes the Anti-Tumor Activity of Xeloda (Capecitabine) in the Metastatic CRC Cell Line

The IC_50_ value of CAP in SW-620 cells was calculated from the dose–response curve of CAP (equal 5 mM) and further it was used in the calculation of the combination index at a fixed ratio with PRO. The combined therapy resulted in a synergistic interaction in SW-620 cells as illustrated in Table 3.

**Figure 8:**
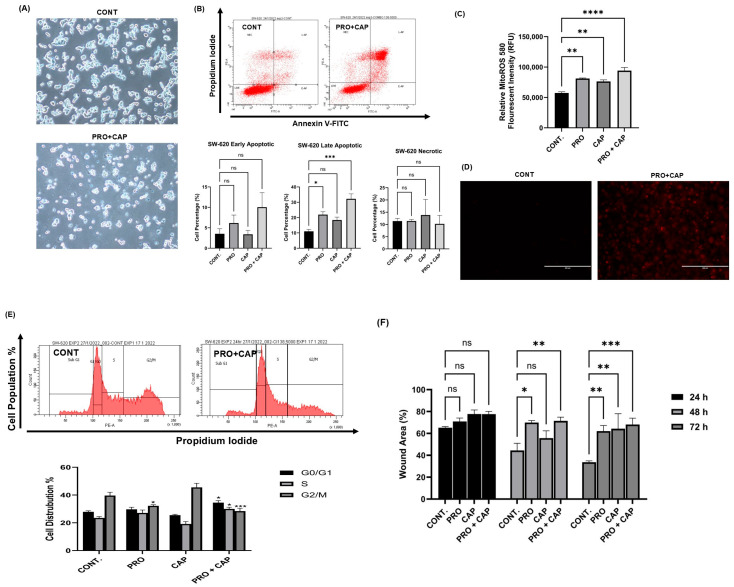
The effects of PRO and CAP combination on the SW-620 metastatic CRC cell line. (**A**) The morphological images of SW-620 after the treatment with combined therapy. Images were taken using an inverted microscope at 10× magnification. (**B**) The detection of the apoptosis in SW-620 after the treatments using Annexin V-FITC/PI assay and flow cytometry. (**C**) Mito ROS generation in SW-620 cells after the treatments using MitoROS 580 dye and a fluorometric technique. (**D**) The fluorescent images of Mito ROS which were captured at a 20× magnification using an EVOS FL fluorescent microscope. (**E**) The flow cytometric analysis of the cell cycle phase distribution after the treatments using PI dye. (**F**) The cell migration assay after the treatments at different time intervals (0, 24, 48, and 72 h). Data are expressed as the mean of three independent experiments ± standard error of the mean (n = 3 ± SEM). Comparisons of means were made using one-way analysis of variance (ANOVA) and two-way ANOVA tests in GraphPad Prism 9.5.1. ns = non-significant value (*p* > 0.05), * *p* ≤ 0.05, ** *p* ≤ 0.01, *** *p* ≤ 0.001, and **** *p* ≤ 0.0001.

As presented in Figure 8A, the microscopic images demonstrated that PRO + CAP treatment clearly causes abnormal changes in the morphology of SW-620 cells such as cell size reduction, loss of adhesion, and formation of apoptotic bodies in comparison to untreated cells. Interestingly, the flow cytometric analysis determined that the combination regimen significantly induced the late apoptosis in SW-620 cells (32.24% versus control 11.03%; *** *p*-value _PRO + CAP_ ≤ 0.001) with a higher score than monotherapies (Figure 8B). PRO activated the late apoptosis (* *p*-value _PRO_ ≤ 0.05 versus control), while the chemotherapy CAP did not activate the apoptosis (*p*-value _CAP_ ˃ 0.05 versus control). Additionally, PRO or CAP monotherapies generated high levels of Mito ROS (** *p*-value _PRO or CAP_ ≤ 0.01 versus control), whereas PRO+ CAP treatment led to the over-generation of Mito ROS (**** *p* ≤ 0.0001 versus control) (Figure 8C). The fluorescent images in Figure 8D showed the greater intensity of the red fluorescence in the combined group versus the control, which implies the greater mitochondrial oxidative stress caused by over-generation of Mito ROS (superoxide), leading to induce the mitochondrial apoptosis in SW-620 cells treated with double therapy. Moreover, the double therapy altered the cell cycle of SW-620 via arresting the cells at the G0/G1 and S phases (* *p* ≤ 0.05 versus control) and decreasing the cell population at the G2/M phase (*** *p* ≤ 0.001 versus control) (Figure 8E). For cells treated with the double therapy, the cell population significantly increased to 34.50% at the G0/G1 phase versus the control (27.90%). At the S phase, the cell population increased to 30.02% versus the control (23.50%), while the cell population at the G2/M phase decreased to 28.43% versus the control (39.60%). However, PRO therapy only decreased the cell population at the G2/M phase (* *p* ≤ 0.05 versus the control), but CAP therapy did not alter the cell cycle of SW-620 cells, which is consistent with the non-apoptotic effect of CAP on SW-620 (Figure 8E). For the analysis of the cell migration assay, the combined therapy caused a significant reduction in the cell migration ability of SW-620 at the 48 and 72 h time intervals in contrast to the control, with higher scores than mono therapies (** *p* ≤ 0.01 and *** *p* ≤ 0.001) (Figure 8F). PRO therapy inhibited the cell migration of SW-620 at the 48 and 72 h time intervals in contrast to control (* *p* ≤ 0.05 and ** *p* ≤ 0.01), but CAP therapy only inhibited the cell migration at 72 h (** *p* ≤ 0.01 versus control). The output of all experiments revealed the synergistic interaction of PRO when coupled with CAP in the metastatic CRC cell line.

## 3. Discussion

CRC is characterized by the hyperactivation of oncogenes (*KRAS*, *BRAF*, and *PIK3CA*) and the loss of tumor suppressor genes (*TP53* and *PTEN*), along with irregularities in multiple signaling pathways [25]. The current study aimed to investigate the anti-tumor activity of PRO as a repurposed drug across various human CRC cell lines in terms of cell cytotoxicity, cell survival, cell migration, and several biochemical parameters.

MTT and Trypan blue assays were used to measure viable cells after PRO treatment. Initially, the Trypan blue assay was used as a primary indication for PRO’s cytotoxicity. An MTT assay was then performed to obtain accurate cytotoxic data. For the Trypan blue assay, the data indicated that PRO therapy decreased cell viability in a cell-type-specific and concentration-dependent manner after 24 h. Using the MTT assay, significant drug screening was conducted to measure the cytotoxicity of PRO in the four CRC cell lines. The results showed that with increasing cell density, the IC_50_ values of PRO therapy gradually increased for each cell line, but over time (at 6, 24, 48, and 72 h), these IC_50_ values markedly decreased. Hence, the MTT data indicated that PRO reduced the proliferation of human CRC cells in a cell-type-specific, time-dependent, and dose-dependent manner.

The reasons behind the varied responses of the cells to PRO treatment might be due to variations in their features, in addition to other genetic and epigenetic factors, such as the mutation of the proto-oncogene (*PIK3CA*), the mutation of the apoptotic gene (*TP53*), microsatellite stability status (MSI), epigenetic alterations, the adrenergic receptor expression level, the type of adrenergic receptor expressed on the cell surface (ADRB 1, 2, or 3), the heterogeneity of cells, tumor origin, and duplicate growth time. In the current study, HCT-116 had high IC_50_ values for PRO, which might be due to the following: it is a Duke’s stage D metastatic carcinoma [13]; it is homogenous; and it has a *mut-K-RAS* oncogene (G13D) [26,27], *mut PIK3CA* (H1047R), epigenetic alterations, high MSI, and the fastest duplication time (18 h) among all cell lines [28]. By contrast, HT-29 had the lowest IC_50_ values, which may be due to the following: non-metastatic adenocarcinoma, Duke’s stage C [13], *mut PIK3CA* (P449T), *wt K-RAS* [27], *mut B-RAF* (V600E), chromosomal instability (*CIN*+) [26,27], MSS, epigenetic alterations, *mut TP53*, and 24 h duplicate time [29]. Even HCT-116 and HT-29 cells had a mutation in the *PIK3CA* gene, but HT-29 cells were more sensitive to PRO than HCT-116 and the other cell lines. SW-480 and SW-620 displayed identical genetic mutation profiles but showed epigenetic differences [27]. Therefore, they exhibited different responses to PRO.

Chin et al. researched human CRC cells and found that exposure to PRO for 72 h significantly diminished the viability of SW-620, Colo-205, and HT-29 cells at IC_50_ values of 119.5, 86.38, and 69.1 µM, respectively, with slight effects on SW-480 and DLD-1 cells, but no notable effect on SW-1116 and HCT-116 cells [13]. The IC_50_ values in this study were relative to our findings at the 48 h time point. In the present study, at the 72 h time point, cell viability was reduced with lower IC_50_ values than those previously reported [13]. Moreover, Coelho et al. (2015) reported that PRO inhibited the growth of HT-29 cells at 24 h with an IC_50_ of 65.4 mM [6]. Another study reported that PRO reduced cell growth in a dose-dependent manner when HCT-116 and HT-29 cells were treated for 36 h at 5 × 10^3^ cells/well with IC_50_ of 25.5 and 39.04 µM, respectively [18]. In addition, a study conducted on triple-negative breast cancer cells (TNBC and MDA-MB-231) found that the IC_50_ for PRO was between 180 and 261.5 µM [10]. Shibuya et al. (2022) performed an in vitro study on human oral squamous cell carcinoma (OSCC) using different PRO concentrations for 24 h and found a significant reduction in OSCC cells at doses higher than 300, 110, and 100 µM for SCC-9, Cal-27, and SCC-25, respectively [16]. These studies agree with our finding that PRO decreases cell viability in a cell-type-specific, time-dependent, and dose-dependent manner. Microscopic images of the PRO-treated cells verified the cytotoxic effects of PRO therapy, which appeared as alterations in cell morphology compared with untreated cells. PRO therapy clearly reduced cell density and caused abnormal changes in cell morphology, such as cell shrinkage and conversion to a round shape, similar to apoptotic cells.

For the clonogenic assay, the examination of cell survival was performed after cells were exposed to PRO for 24 h, and then the PRO-treated cells were further incubated with drug-free media for the indicated time, which could be as long as a week, differing from previous studies that have examined colony formation upon long exposure to treatments (10–12 days) [30,31]. The results proved that PRO therapy markedly inhibited colony formation, even after replacing the drug-free media. This revealed that altering the cell’s ability to form colonies, even in the absence of a drug and maintained under normal culture conditions, could be due to the prolonged effects of PRO treatment on cell features (cell survival). Moreover, this effect was observed in all cells, even in those with a low response to PRO (HCT-116).

Regarding the cell migration point, both the *KRAS* and *PIK3CA* mutations promote cell migration, leading to aggressive tumor growth and metastasis. However, *PIK3CA* mutations are more likely to promote migration than *KRAS* mutations. The *PIK3CA* (H1047R) variant is one of the most frequent PIK3CA variants detected in metastatic CRC, accounting for 9.8% of cases [32]. Although the examined CRC cell lines have mutations in *K-RAS*, *B-RAF*, and *PIK3CA*, their migration ability was inhibited to varying extents when exposed to PRO in a cell-type-specific and time-dependent manner. Furthermore, the variation in wound closure after PRO therapy might be related to genetic/epigenetic differences in the CRC cell lines. In this study, the SW-480 cell line (*mut K-RAS*) migration [27] was inhibited more by PRO than by other cells, suggesting that *PIK3CA* plays a major role in controlling migration compared with *K-RAS*. HCT-116 cells have a mutated *PIK3CA* (H1047R) [27], and the data showed that the migration capacity was restored at 72 h (with no significant differences in wound area % compared with the control). By contrast, HT-29 cells have a mutated *PIK3CA* (P449T) [27] variant that cannot be detected in metastatic CRC [32]. Moreover, HT-29 contains *wt K-RAS* and *mut B-RAF* (V600E) [27]. Therefore, these findings support the importance of precision medicine in selecting effective therapies for CRC. Regarding the colony formation assay and cell migration, several reports show that PRO can inhibit colony formation and migration in different cell types, including CRC cells. It has also been reported to inhibit colony formation in tumor cells, including HCT-116 cells [31,33]. Furthermore, cell proliferation and migration are inhibited by PRO in CRC cells (HCT-116 and HT-29) [18,19,33,34] and different cell types [16,33,35]. Our findings clearly demonstrate that PRO therapy can reduce cell migration and colony formation, which agrees with the aforementioned studies.

In addition, the biochemical assays showed that PRO modified these parameters in CRC cells in a cell-type-specific and dose-dependent manner. These results indicate that all CRC cells underwent apoptosis when exposed to PRO. Cell cycle experiments demonstrated that cells were arrested in the G0/G1 phase in HCT-116 and HT-29. In addition, the cell cycle of SW-620 was arrested at the S phase. The analysis of the intracellular ROS assay revealed that only HT-29 cells generated higher levels of intracellular ROS, while other cells reduced the formation of ROS in contrast to the control. The Mito ROS assay revealed that all examined CRC cells produced high levels of Mito ROS except HCT-116. Furthermore, the scavenging of intracellular ROS during PRO treatment may be related to drug resistance [36]. The findings of the current study showed that apoptosis activation in HT-29 cells could be mediated via a mitochondrial pathway coupled with oxidative stress through the over-generation of intracellular and Mito ROS. By contrast, apoptosis in SW-480 and SW-620 cells might occur via a mitochondrial pathway through Mito ROS generation. By contrast, apoptosis in HCT-116 cells did not occur through oxidative stress or the mitochondrial pathway. These results reveal that HT-29 is the most sensitive CRC cell line to PRO treatment, even with mutations in the *PIK3CA*, *TP53*, and *B-RAF* (V600E) genes. A recent study reported that a 48 h PRO treatment activated ferroptosis oxidative cell death in HT-29 cells via the accumulation of intracellular and Mito ROS [19]. Moreover, a previous study determined that PRO can cause apoptosis by inducing both the extrinsic and mitochondrial pathways in melanoma cells [14], which agrees with our findings.

Regarding CRC cell death, cell cycle, and ROS measurements, only a few reports demonstrate that this agent can induce apoptosis, cell cycle arrest, and ROS generation in different cell types, including CRC cells. Chin et al. demonstrated that exposure to PRO increased the number of apoptotic cells and arrested the cell cycle at the G1 phase in wild-type *K-RAS* human CRC cell lines (HT-29 and Colo-205 cells) [13]. By contrast, our findings indicated that PRO arrested the cell cycle at the G1 phase in the *wt K-RAS* CRC cell line (HT-29) and the *mut K-RAS* CRC cell line (HCT-116) and arrested cells in the S phase in *mut K-RAS* (SW-620). Anselmino et al. reported that PRO did not markedly induce apoptosis in HCT-116 cells [18]. Another study showed that PRO treatment increased the populations of apoptotic and necrotic cells in HCT-116 spheroid cell culture conditions [33]. However, in this study, PRO induced apoptosis in HCT-116 cells, with no necrotic response. PRO induces apoptosis and cell cycle arrest at the G0/G1 and G2/M phases in human gastric adenocarcinoma cell lines (SGC-7901 and BGC-823) [37]. In addition, PRO activates apoptosis and arrests the cell cycle at the G0/G1/S phase in melanoma cells [38]. Zhao et al. reported that PRO induced apoptosis, cell cycle arrest at the G2/M phase, and increased intracellular ROS levels via activation of the ROS/JNK signaling pathway in human ovarian cancer cells [14]. A recent study showed that PRO increased Mito ROS levels in isolated mitochondria (in vitro) [39], which agrees with the PRO effects observed in our study in HT-29, SW-480, and SW-620 cells.

The current findings demonstrate that PRO can induce apoptosis, alter the cell cycle, and change intracellular and mitochondrial ROS levels; to a certain extent, this agrees with the previously mentioned studies, depending on the cell type, drug dosage, and culture conditions. Moreover, PRO was more potent in inducing apoptosis, cell cycle, and ROS in HT-29 cells than in the other examined CRC cell lines, supporting that genetic/epigenetic profiles and characterizations of different cells may exhibit different sensitivities to PRO. Furthermore, PRO was more potent in inhibiting the migration of the SW-480 cell line than the other CRC cell lines examined.

Moreover, our findings are not limited to the application of PRO monotherapy but they also demonstrate that combining PRO with CAP enhances the anti-cancer effects on SW-620 metastatic CRC cells. This dual therapy induced mitochondrial apoptosis, which agrees with our previous findings in HT-29 cells [19]. In addition, it altered the cell cycle and inhibited the migration of SW-620 cells. The synergy observed may be attributed to PRO’s modulation of apoptosis, Mito ROS generation, and cell cycle alteration [19,37,39].

## 4. Materials and Methods

### 4.1. Cell Culture

Human CRC cell lines (HCT-116, HT-29, SW-480, and SW-620) were provided by King Faisal Specialist Hospital and Research Center, Jeddah branch (KFSHRC-J), Jeddah, Kingdom of Saudi Arabia (KSA). Cells were cultured in DMEM high-glucose media supplemented with 10% fetal bovine serum (FBS) and penicillin/streptomycin (1%) (Beijing Solarbio Science & Technology Co., Ltd., Beijing, China).

### 4.2. Propranolol Therapy

Stock solution of propranolol hydrochloride (Sigma-Aldrich, St. Louis, MO, USA) was prepared in PBS at a concentration of 1 mg/mL and then filtered and kept at −20 °C until use. Serial dilutions of PRO were freshly prepared on the day of use by diluting the PRO stock solution in appropriate volumes of culture medium (320, 160, 80, 40, 20, 10, 5, and 2.5 µM) [9]. Serial dilutions of PRO were used in the Trypan blue exclusion assay and the 3-(4,5-Dimethylthiazol-2-yl)-2,5-diphenyltetrazolium bromide (MTT) assay to measure its cytotoxicity in each CRC cell line.

### 4.3. Trypan Blue Exclusion Assay

CRC cells were seeded in 24-well plates at a density of 50 × 10^3^ cells/well and incubated for 24 h at 37 °C in 5% CO_2_. The culture medium was then replaced with serial dilutions of PRO as the treatment group, and growth medium was used as the control group. The cells were then incubated for 24 h. After collecting the cells, they were stained with Trypan blue dye (0.4%) (Gibco, Thermo Fisher Scientific, Waltham, MA, USA), the cell suspension mixture (1:1) in each group was applied to the hemocytometer, and the viable cells (unstained) were counted using a Nikon inverted microscope (Nikon Eclipse, Tokyo, Japan) [38]. The percentage of viable cells was calculated using the following equation.

$$Viable\;cells\;\%=\frac{Total\;number\;of\;viable\;cells\;in\;treated\;group}{Total\;number\;of\;viable\;cells\;in\;untreated\;group}\times 100$$

This test was performed in triplicate for each cell line (n = 3).

### 4.4. MTT Assay

To study the cytotoxicity of PRO, the viability of CRC cells was assessed using the MTT assay (Invitrogen, Thermo Fisher Scientific, Waltham, MA, USA) at various cell densities and over time following PRO therapy [40].

#### 4.4.1. Cell Viability at Various Cell Densities

Cells were seeded in 96-well plates at different cell densities (2, 5, 10, 15, and 20 × 10^3^ cells/well) in separate experiments for each density. CRC cell lines were incubated overnight at 37 °C in a 5% CO_2_ atmosphere. The following day, the growth medium was replaced with serial dilutions of PRO for 24 h (constant time at different cell densities). After treatment, the MTT assay was performed as previously described [40]. The absorbance of the purple color was measured using a SpectraMax i3 microplate reader (Molecular Devices, LLC, San Jose, CA, USA) at 570 nm. Each condition was repeated three times for all the examined cell lines (n = 3).

#### 4.4.2. Cell Viability over Time Intervals

Cell viability was measured over time using the MTT assay. Cells were seeded at constant cell density (10 × 10^3^ cells/well) in 96-well plates as a separate experiment for each time point and incubated for 24 h at 37 °C in 5% CO_2_. The next day, the medium was replaced with serial dilutions of PRO and the cells were incubated for 6, 24, 48, and 72 h. At the end of the incubation period, the MTT assay was conducted as described. Each condition was repeated thrice for all cell lines (n = 3).

### 4.5. Morphological Analysis

In 6-well plates, HCT-116 and SW-620 cells were seeded at a density of 2 × 10^5^ cells/well, whereas a 3 × 10^5^ cells/well density was used for HT-29 and SW-480. CRC cells were incubated for 24 h at 37 °C and 5% CO_2_. For each cell line, the medium was replaced with PRO (IC_50_) and the cells were incubated for 24 h. Following treatment, morphological images were taken using a Nikon Eclipse microscope (Nikon Eclipse, Tokyo, Japan) at 10× magnification [19].

### 4.6. Colony Formation Assay

To assess the ability of the CRC cells to form colonies after PRO treatment, a clonogenic assay was performed [41]. Briefly, HCT-116 cells were plated at a density of 500 cells/well in 6-well plates. HT-29, SW-480, and SW-620 cells were plated at 1000 cells/well in 6-well plates. Following treatment with PRO for 24 h, the cells were cultured in drug-free medium until control cells formed approximately 100 colonies. HCT-116 cells were incubated for 3 days, whereas HT-29, SW-480, and SW-620 were incubated for 7–9 days in 5% CO_2_ at 37 °C. The colonies were then rinsed twice with PBS and fixed with 70% ethanol for 10–15 min at room temperature. Colonies were stained with aqueous crystal violet dye (0.1% W/V) (Sigma-Aldrich, St. Louis, MO, USA) for 10–15 min and washed twice with deionized water. The number of colonies was counted using an inverted light microscope (Nikon Eclipse, Tokyo, Japan) at a magnification of 10×, and images were taken using a camera. The cell survival assay was performed in triplicate. The treated colonies were counted and compared with untreated colonies.

### 4.7. Detection of Cell Death Mechanism

The Annexin V-FITC/PI assay (ab14085, Abcam, Cambridge, UK) was used to determine whether the cytotoxic effect of PRO could induce cell death (early and late apoptosis and/or necrosis) in the treated cells compared with the control [42]. Briefly, the cells were seeded in 6-well plates, as described in Section 4.5. After treatment, cells were collected and washed with PBS. The experiment was carried out according to the Annexin V-FITC/PI kit manufacturer’s instructions. Subsequently, analysis was performed using a BD FACSCantoTM II flow cytometry instrument (BD Biosciences, Franklin Lakes, NJ, USA), and a minimum of 10,000 events were acquired.

### 4.8. Cell Cycle Analysis

The distribution of cell cycle phases was analyzed to determine whether inducing cell death after PRO treatment could alter the cell cycle phases in contrast to those of control cells [38]. CRC cells were seeded in 6-well plates as described in Section 4.5. Following treatment, the cells were collected and washed. The cells were then fixed with 2 mL of ice-cold fixation buffer (70% ethanol) for 24 h under cold conditions. After fixation, cells were centrifuged and washed twice. Propidium iodide (PI) (BD Biosciences, Franklin Lakes, NJ, USA) staining was performed according to the manufacturer’s instructions. DNA content was measured in each phase of the cell cycle using a BD FACSAria^TM^ III flow cytometer (BD Biosciences, Franklin Lakes, NJ, USA). At least 10,000 events were acquired in the list mode.

### 4.9. Measurement of Intracellular ROS Level

The aim of this experiment was to determine whether PRO can generate ROS in CRC cell lines using the H2DCFDA kit (ab113851, Abcam, Cambridge, UK) and flow cytometry [43]. Cells were seeded in 12-well plates at a density of 7 × 10^4^ cells/well for HCT-116 and SW-620 or at a density of 1 × 10^5^ cells/well for HT-29 and SW-480. After treatment, staining with H2DCFDA dye was performed according to the manufacturer’s instructions. The fluorescence intensity of DCF was measured using a BD FACSAria III flow cytometer, and a minimum of 20,000 events were recorded. For intracellular ROS fluorescent imaging, the DCFH2-DA kit was used according to the manufacturer’s instructions. Images were captured using an EVOS FL (Life Technologies, Carlsbad, CA, USA) fluorescence microscope.

### 4.10. Measurement of Mitochondrial Reactive Oxygen Species (Mito ROS) Level

We further examined whether apoptosis was mediated by mitochondria via excess formation of Mito ROS using the MitoROS 580 kit (ab219943, Abcam, Cambridge, UK) [44]. Briefly, 1 × 10^4^ cells/well of CRC cell lines were seeded in 96-well plates and incubated overnight in 5% CO_2_ at 37 °C. After treatment, the MitoROS 580 assay was performed according to the manufacturer’s instructions. Red fluorescence intensity was directly assessed using a SpectraMax i3 fluorometric microplate reader (Molecular Devices, LLC, USA), with excitation and emission wavelengths of 540 nm and 590 nm, respectively. A proper protocol for fluorescence imaging was performed as described in the MitoROS 580 kit. Images were captured using the EVOS FL fluorescence microscope.

### 4.11. In Vitro Migration Assay

The migration ability of CRC cell lines after PRO therapy was determined using in vitro wound healing and scratch assays [45]. Briefly, cells were plated as described in Section 4.5 and incubated for 24 h. Following attachment, a wound scratch line was made across the middle of the well with a sterile 200 μL pipette tip. After scratching, the medium was changed and the wells were washed with the new medium to remove cells (debris). Subsequently, the treatment was applied directly to the cells and the plates were incubated in 5% CO_2_ at 37 °C. Images were captured with a microscope at a magnification of 10× using an inverted microscope at different time intervals (0, 24, 48, and 72 h). The measurement of the wound area % in the captured images was analyzed using the Fiji software (ImageJ version 2.9.0) (https://imagej.net/software/fiji/ accessed on 20 January 2023).

### 4.12. The Combination Therapy

To evaluate potential synergy, SW-620 cells were treated with various concentrations of CAP and PRO, alone and in combination, for 24 h. Firstly, IC_50_ values for CAP was determined and fixed-ratio combinations were applied. The type of drug interaction in the combination was determined by the Chou–Talalay method [46], which is the standard measure for quantifying the potential drug synergism. The IC_50_ values of PRO and CAP obtained from the dose–response curves by GraphPad Prism version 9.0 were used for the double therapy. Then, the combination index values (CI) were calculated by using the CompoSyn 1.0 software (www.combosyn.com, accessed on 23 July 2022), which depends on the Chou–Talalay theorem. Briefly, a total of 10 × 10^3^ cells/well were seeded in 96-well plates. The following day, the cells were incubated with PRO and/or CAP at five concentrations (0.25 × IC_50_, 0.5 × IC_50_, 1 × IC_50_, 2 × IC_50_, and 4 × IC_50_) for 24 h. The cell viability of combination was measured using the MTT assay. The CI test was carried out in triplicate (n = 3). Moreover, we performed several assays to determine the impact of the dual therapy on the apoptosis induction, cell cycle, Mito ROS, and cell migration as outlined above in each related section.

### 4.13. Statistical Analysis

The data are expressed as the mean ± standard error of the mean (SEM) of at least three independent replicates (n = 3) for all CRC cell lines. GraphPad Prism version 9.5.1 (GraphPad Software Inc., San Diego, CA, USA) was used to calculate the IC_50_ values of PRO and CAP from the dose–response curves, comparing the differences between PRO and the control using the unpaired Student’s *t*-test in GraphPad Prism. The differences between more than two groups were calculated using one-way analysis of variance (ANOVA) followed by Dunnett’s test in GraphPad Prism. Two-way ANOVA followed by Tukey’s test was used to compare the mean differences in two variables between three or more groups. The CI values were calculated using the CompuSyn version 1.0 software (Combusyn, Inc., Paramus, NJ, USA). Fiji (ImageJ version 2.9.0) (https://imagej.net/software/fiji/) was used with the wound healing tool size plugin (https://imagej.net/plugins/ accessed on 20 January 2023) to analyze wound images. The significance level in all cases was *p*-value ≤ 0.05.

## 5. Conclusions

Examination of PRO underscores its potential as a valuable therapeutic option for addressing both primary and advanced CRC. This study is significant because it evaluates the efficacy of PRO treatment on human CRC cell lines under diverse experimental conditions, including different cell lines, increasing cell densities, and several time points. These findings are foundational references for future studies. Notably, our research offers insights into the inhibition of colony formation and anti-migration potential of PRO treatment in an in vitro setting. The data demonstrated that adding PRO to CRC cell lines (HCT-116, HT-29, SW-480, and SW-620) had differential effects on cytotoxicity, colony formation, apoptosis, cell cycle progression, intracellular ROS, mitochondrial ROS, and cell migration. This study revealed the significant anticancer effects of this regimen in preclinical models of advanced CRC. Therefore, the use of primary CRC cell lines may enhance their anticancer properties. Examination of preclinical models of CRC with non-mutated states of certain proto-oncogenes and tumor suppressor genes could have additional impacts. In addition, the in vitro synergy observed in PRO + CAP dual therapy highlighted the potential therapeutic benefits in treating metastatic CRC and further in vivo investigation is required.

## Figures and Tables

**Table 1:** IC_50_ values of PRO (µM) in CRC cell lines at different cell densities (2, 5, 10, 15, and 20 × 10^3^ cells/well) at 24 h. Data are expressed as the mean ± standard error of three independent experiments (n = 3).

Cell Line	Cell Densities				
	2 × 10^3^	5 × 10^3^	10 × 10^3^	15 × 10^3^	20 × 10^3^
HCT-116	44.73 ± 7.37	79.49 ± 11.26	166.08 ± 1.31	203.80 ± 47.57	270.86 ± 42.27
HT-29	28.14 ± 4.73	43.79 ± 12.22	131.16 ± 6.70	157.73 ± 7.92	161.16 ± 5.60
SW-480	54.50 ± 15.48	104 ± 1.15	130.87 ± 8.75	135.40 ± 3.26	160.96 ± 12.35
SW-620	20.75 ± 5.71	21.55 ± 3.49	138.03 ± 22.35	234.50 ± 8.38	219.66 ± 26.50

**Table 2:** IC_50_ values of PRO (µM) for CRC cell lines over different time intervals (6, 24, 48, and 72 h) at a cell density of 10 × 10^3^ cells/well. The data are expressed as the mean ± standard error of three independent experiments (n = 3).

Cell Line	6 h	24 h	48 h	72 h
HCT-116	303.95 ± 3.49	163.80 ± 14.78	129.26 ± 5.45	95.86 ± 13.11
HT-29	225.60 ± 4.85	135.60 ± 2.40	75.71 ± 12.58	53.53 ± 6.79
SW-480	176.60 ± 37.35	131.24 ± 0.66	88.66 ± 14.14	67.16 ± 12.86
SW-620	208.06 ± 10.67	150.53 ± 11.54	122.10 ± 2.85	94.67 ± 11.16

**Table 3:** The combination index (CI) values of the double therapy (PRO and CAP) for the SW-620 cell line at the median effective doses (ED50, 75, 90, and 95). The CI values were obtained from CompuSyn software. The CI theorem interpretates the drug interaction as: synergy (CI < 1), additivity (CI = 1), or antagonism (CI > 1).

The Median Effective Doses (ED)	CI (ED50)	CI (ED75)	CI (ED90)	CI (ED95)	Type of Interaction
SW-620	0.73	0.75	0.77	0.79	Synergism

## Data Availability

The datasets used and/or analyzed during the current study are available from the corresponding author upon reasonable request. The compounds and cell lines used in this study are commercially available.

## References

[1] Pantziarka P., Bouche G., Sukhatme V., Meheus L., Rooman I., Sukhatme V.P. (2016). Repurposing Drugs in Oncology (ReDO)—Propranolol as an Anti-Cancer Agent. Ecancermedicalscience.

[2] Albiñana V., Gallardo-Vara E., Casado-Vela J., Recio-Poveda L., Botella L.M., Cuesta A.M. (2022). Propranolol: A “Pick and Roll” Team Player in Benign Tumors and Cancer Therapies. J. Clin. Med..

[3] Hoeger P.H., Harper J.I., Baselga E., Bonnet D., Boon L.M., Di Chiara Atti M., El Hachem M., Oranje A.P., Rubin A.T., Weibel L. (2015). Treatment of Infantile Haemangiomas: Recommendations of a European Expert Group. Eur. J. Pediatr..

[4] Verbaanderd C., Meheus L., Huys I., Pantziarka P. (2017). Repurposing Drugs in Oncology: Next Steps. Trends Cancer.

[5] Nowak-Sliwinska P., Scapozza L., Ruiz i Altaba A. (2019). Drug Repurposing in Oncology: Compounds, Pathways, Phenotypes and Computational Approaches for Colorectal Cancer. Biochim. Biophys. Acta Rev. Cancer.

[6] Coelho M., Moz M., Correia G., Teixeira A., Medeiros R., Ribeiro L. (2015). Antiproliferative Effects of β-Blockers on Human Colorectal Cancer Cells. Oncol. Rep..

[7] Wolter J.K., Wolter N.E., Blanch A., Partridge T., Cheng L., Morgenstern D.A., Podkowa M., Kaplan D.R., Irwin M.S. (2014). Anti-Tumor Activity of the Beta-Adrenergic Receptor Antagonist Propranolol in Neuroblastoma. Oncotarget.

[8] Chang P.Y., Huang W.Y., Lin C.L., Huang T.C., Wu Y.Y., Chen J.H., Kao C.H. (2015). Propranolol Reduces Cancer Risk: A Population-Based Cohort Study. Medicine.

[9] Wang F., Liu H., Wang F., Xu R., Wang P., Tang F., Zhang X., Zhu Z., Lv H., Han T. (2018). Propranolol Suppresses the Proliferation and Induces the Apoptosis of Liver Cancer Cells. Mol. Med. Rep..

[10] Xie W.Y., He R.H., Zhang J., He Y.J., Wan Z., Zhou C.F., Tang Y.J., Li Z., McLeod H.L., Liu J. (2019). β-Blockers Inhibit the Viability of Breast Cancer Cells by Regulating the ERK/COX-2 Signaling Pathway and the Drug Response Is Affected by ADRB2 Single Nucleotide Polymorphisms. Oncol. Rep..

[11] Liao P., Song K., Zhu Z., Liu Z., Zhang W., Li W., Hu J., Hu Q., Chen C., Chen B. (2020). Propranolol suppresses the growth of colorectal cancer through simultaneously activating autologous CD8+ T cells and inhibiting tumor AKT/MAPK pathway. Clin. Pharmacol. Ther..

[12] Lin Q., Wang F., Yang R., Zheng X., Gao H., Zhang P. (2013). Effect of chronic restraint stress on human colorectal carcinoma growth in mice. PLoS ONE.

[13] Chin C.C., Li J.M., Lee K.F., Huang Y.C., Wang K.C., Lai H.C., Cheng C.C., Kuo Y.H., Shi C.S. (2016). Selective β2-AR blockage suppresses colorectal cancer growth through regulation of EGFR-Akt/ERK1/2 signaling, G1-phase arrest, and apoptosis. J. Cell Physiol..

[14] Zhao S., Fan S., Shi Y., Ren H., Hong H., Gao X., Zhang M., Qin Q., Li H. (2020). Propranolol induced apoptosis and autophagy via the ROS/JNK signaling pathway in human ovarian cancer. J. Cancer.

[15] Zhang L., Mai H.M., Zheng J., Zheng J.W., Wang Y.A., Qin Z.P., Li K.L. (2014). Propranolol inhibits angiogenesis via down-regulating the expression of vascular endothelial growth factor in hemangioma derived stem cell. Int. J. Clin. Exp. Pathol..

[16] Shibuya C.M., Tjioe K.C., Oliveira S.H.P., Bernabé D.G. (2022). Propranolol inhibits cell viability and expression of the pro-tumorigenic proteins Akt, NF-ĸB, and VEGF in oral squamous cell carcinoma. Arch. Oral Biol..

[17] Fjæstad K.Y., Rømer A.M.A., Goitea V., Johansen A.Z., Thorseth M.L., Carretta M., Engelholm L.H., Grøntved L., Junker N., Madsen D.H. (2022). Blockade of beta-adrenergic receptors reduces cancer growth and enhances the response to anti-CTLA4 therapy by modulating the tumor microenvironment. Oncogene.

[18] Anselmino L.E., Baglioni M.V., Malizia F., Laluce N.C., Etichetti C.B., Marignac V.L., Rozados V., Scharovsky O.G., Girardini J., Rico M.J. (2021). Repositioning metformin and propranolol for colorectal and triple negative breast cancers treatment. Sci. Rep..

[19] Alzahrani S.M., Al Doghaither H.A., Alkhatabi H.A., Basabrain M.A., Pushparaj P.N. (2025). Propranolol and Capecitabine Synergy on Inducing Ferroptosis in Human Colorectal Cancer Cells: Potential Implications in Cancer Therapy. Cancers.

[20] Mueller M., Schneider M.A., Deplazes B., Cabalzar-Wondberg D., Rickenbacher A., Turina M. (2021). Colorectal cancer of the young displays distinct features of aggressive tumor biology: A single-center cohort study. World J. Gastrointest. Surg..

[21] Alzahrani S.M., Al Doghaither H.A., Al Ghafari A.B. (2021). General insight into cancer: An overview of colorectal cancer. Mol. Clin. Oncol..

[22] Zhang Z., Bao C., Jiang L., Wang S., Wang K., Lu C., Fang H. (2023). When cancer drug resistance meets metabolomics (bulk, single-cell and/or spatial): Progress, potential, and perspective. Front. Oncol..

[23] Ricon-Becker I., Haldar R., Shabat Simon M., Gutman M., Cole S.W., Ben-Eliyahu S., Zmora O. (2023). Effect of perioperative COX-2 and beta-adrenergic inhibition on 5-year disease-free-survival in colorectal cancer: A pilot randomized controlled Colorectal Metastasis PreventIon Trial (COMPIT). Eur. J. Surg. Oncol..

[24] Peixoto R., Pereira M.D.L., Oliveira M. (2020). Beta-blockers and cancer: Where are we?. Pharmaceuticals.

[25] Dosunmu G.T., Shergill A. (2024). Colorectal cancer: Genetic underpinning and molecular therapeutics for precision medicine. Genes.

[26] Hinoue T., Weisenberger D.J., Pan F., Campan M., Kim M., Young J., Whitehall V.L., Leggett B.A., Laird P.W. (2009). Analysis of the association between CIMP and BRAF in colorectal cancer by DNA methylation profiling. PLoS ONE.

[27] Ahmed D., Eide P.W., Eilertsen I.A., Danielsen S.A., Eknæs M., Hektoen M., Lind G.E., Lothe R.A. (2013). Epigenetic and genetic features of 24 colon cancer cell lines. Oncogenesis.

[28] Schulte Am Esch J., Windmöller B., Hanewinkel J., Storm J., Förster C., Wilkens L., Krüger M., Kaltschmidt B., Kaltschmidt C. (2020). Isolation and characterization of two novel colorectal cancer cell lines, containing a subpopulation with potential stem-like properties: Treatment options by MYC/NMYC inhibition. Cancers.

[29] Forgue-Lafitte M.E., Coudray A.M., Bréant B., Mester J. (1989). Proliferation of the human colon carcinoma cell line HT29: Autocrine growth and deregulated expression of the c-myc oncogene. Cancer Res..

[30] Sim J.J., Park M.H., Baek J.H., Lee H., Jeong K.Y., Kim H.M. (2018). Investigation into enhancing capecitabine efficacy in colorectal cancer by inhibiting focal adhesion kinase signaling. Anticancer Res..

[31] Sidorova M., Petrikaitė V. (2022). The effect of beta adrenoreceptor blockers on viability and cell colony formation of non-small cell lung cancer cell lines A549 and H1299. Molecules.

[32] Wang H., Tang R., Jiang L., Jia Y. (2024). The role of PIK3CA gene mutations in colorectal cancer and the selection of treatment strategies. Front. Pharmacol..

[33] Barathova M., Grossmannova K., Belvoncikova P., Kubasova V., Simko V., Skubla R., Csaderova L., Pastorek J. (2020). Impairment of hypoxia-induced CA IX by beta-blocker propranolol—Impact on progression and metastatic potential of colorectal cancer cells. Int. J. Mol. Sci..

[34] Işeri O.D., Sahin F.I., Terzi Y.K., Yurtcu E., Erdem S.R., Sarialioglu F. (2014). Beta-adrenoreceptor antagonists reduce cancer cell proliferation, invasion, and migration. Pharm. Biol..

[35] Zhang T., Qian Y., Yuan C., Wu Y., Qian H., Lu H., Hu C., Li W. (2021). Propranolol suppresses proliferation and migration of HUVECs through regulation of the miR-206/VEGFA axis. BioMed Res. Int..

[36] Shah M.A., Rogoff H.A. (2021). Implications of reactive oxygen species on cancer formation and its treatment. Semin. Oncol..

[37] Liao X., Che X., Zhao W., Zhang D., Long H., Chaudhary P., Li H. (2010). Effects of propranolol in combination with radiation on apoptosis and survival of gastric cancer cells in vitro. Radiat. Oncol..

[38] Bustamante P., Miyamoto D., Goyeneche A., de Alba Graue P.G., Jin E., Tsering T., Dias A.B., Burnier M.N., Burnier J.V. (2019). Beta-blockers exert potent anti-tumor effects in cutaneous and uveal melanoma. Cancer Med..

[39] Seydi E., Tabbati Y., Pourahmad J. (2020). Toxicity of atenolol and propranolol on rat heart mitochondria. Drug Res..

[40] Zarewa S.A., Binobaid L., Sulaiman A.A.A., Sobeai H.M.A., Alotaibi M., Alhoshani A., Isab A.A. (2023). Synthesis, characterization, and anticancer activity of phosphanegold(I) complexes of 3-thiosemicarbano-butan-2-one oxime. Biomedicines.

[41] Li J., Zhu P., Chen Y., Zhang S., Zhang Z., Zhang Z., Wang Y., Jiang X., Lin K., Wu W. (2022). Isoalantolactone induces cell cycle arrest, apoptosis and autophagy in colorectal cancer cells. Front. Pharmacol..

[42] Kumar R., Saneja A., Panda A.K. (2021). An Annexin V-FITC-Propidium Iodide-Based method for detecting apoptosis in a non-small cell lung cancer cell line. Methods Mol. Biol..

[43] Bode K., Link C., Krammer P.H., Weyd H. (2020). Flow-cytometric detection of low-level reactive oxygen species in cell lines and primary immune cells. Bio Protoc..

[44] Kimura K., Chun J.H., Lin Y.L., Liang Y.C., Jackson T.L., Huang R.C.C. (2023). Tetra-O-Methyl-Nordihydroguaiaretic acid inhibits energy metabolism and synergistically induces anticancer effects with temozolomide on LN229 glioblastoma tumors implanted in mice while preventing obesity in normal mice that consume high-fat diets. PLoS ONE.

[45] Miere F., Teuşdea A.C., Laslo V., Cavalu S., Fritea L., Dobjanschi L., Zdrinca M., Ganea M., Paşca C., Pașc P. (2021). Evaluation of in vitro wound-healing potential, antioxidant capacity, and antimicrobial activity of *Stellaria media* (L.) Vill. Appl. Sci..

[46] Chou T.C., Talalay P. (1984). Quantitative Analysis of Dose-Effect Relationships: The Combined Effects of Multiple Drugs or Enzyme Inhibitors. Adv. Enzym. Regul..

